# Comparison of the Detection Characteristics of Trace Species Using Laser-Induced Breakdown Spectroscopy and Laser Breakdown Time-of-Flight Mass Spectrometry

**DOI:** 10.3390/s150305982

**Published:** 2015-03-11

**Authors:** Zhenzhen Wang, Yoshihiro Deguchi, Junjie Yan, Jiping Liu

**Affiliations:** 1 State Key Laboratory of Multiphase Flow in Power Engineering, Xi’an Jiaotong University, No.28, Xianning West Road, Xi’an 710049, China; E-Mails: zhenzhen@stu.xjtu.edu.cn (Z.W.); yanjj@mail.xjtu.edu.cn (J.Y.); liujp@mail.xjtu.edu.cn (J.L.); 2 Graduate School of Advanced Technology and Science, the University of Tokushima, 2-1, Minamijyosanjima, Tokushima 770-8506, Japan

**Keywords:** laser-induced breakdown spectroscopy (LIBS), laser breakdown time-of-flight mass spectrometry (LB-TOFMS), low pressure, electron impact ionization

## Abstract

The rapid and precise element measurement of trace species, such as mercury, iodine, strontium, cesium, *etc.* is imperative for various applications, especially for industrial needs. The elements mercury and iodine were measured by two detection methods for comparison of the corresponding detection features. A laser beam was focused to induce plasma. Emission and ion signals were detected using laser-induced breakdown spectroscopy (LIBS) and laser breakdown time-of-flight mass spectrometry (LB-TOFMS). Multi-photon ionization and electron impact ionization in the plasma generation process can be controlled by the pressure and pulse width. The effect of electron impact ionization on continuum emission, coexisting molecular and atomic emissions became weakened in low pressure condition. When the pressure was less than 1 Pa, the plasma was induced by laser dissociation and multi-photon ionization in LB-TOFMS. According to the experimental results, the detection limits of mercury and iodine in N_2_ were 3.5 ppb and 60 ppb using low pressure LIBS. The mercury and iodine detection limits using LB-TOFMS were 1.2 ppb and 9.0 ppb, which were enhanced due to different detection features. The detection systems of LIBS and LB-TOFMS can be selected depending on the condition of each application.

## 1. Introduction

Recently, various pollution incidents, such as the heavy metal pollutions of lead, cadmium, chromium, mercury, arsenic, *etc.* have resulted in serious influences on the environment and human health. Environmental pollution is becoming increasingly severe and much more attention should be paid to the issue [1,2,3]. Radioactive materials released from nuclear power plant accidents, nuclear plant waste, medical sources and others have also been serious pollution sources in recent years. For example, in Japan, after the Fukushima nuclear power plant accident, the pollution from radioactive materials has become a focal issue and research point. Human and other organisms potentially take up the radioactive elements, such as iodine, cesium, strontium and so on, which leads to severe disease.

On the other hand, in industry there is much demand for research into the integrated coal gasification fuel cell combined cycle (IGFC) to evaluate trace heavy metals. With the development of the combined power generation technologies, IGFC has a high fuel-to-electricity conversion efficiency of 45%–60% compared with conventional thermal power plants with their energy conversion efficiencies of 30%–40% [4,5]. However, fuel impurities in these practical fuels could cause degradation of cell performance and affect system durability. The various fuel impurities in parts per billion (ppb) levels, including sulfur compounds, chlorine and many trace species contained in coal, such as arsenic, phosphorus, antimony, cadmium, mercury and lead, can have adverse effects on the solid oxide fuel cell (SOFC) anode [6,7]. Therefore it is necessary to develop the analytical methods for these impurities to evaluate and control the poisoning effects in SOFCs. Meanwhile, various standards and regulations limiting heavy metal concentrations have been proposed and defined by the WHO, China, and other countries and regions in different applications. For example, in China the limit for mercury emissions from thermal power plants is 0.03 mg/m^3^ (3.35 ppb) (NTP) [8,9]. For the reasons set forth above the target concentration levels are set at ppb based on the real-time measurement needs of these industries.

Due to the low concentration of trace species, enrichment and separation are essential to analyze the content and concentration using conventional methods [10,11,12,13]. These procedures usually take a long time and these methods can’t meet the requirements of some applications. Inductively coupled plasma-atomic emission spectroscopy (ICP-AES) and inductively coupled plasma-mass spectrometry (ICP-MS) have been applied to detect the trace species with high detection ability in various applications [14,15,16,17]. ICP-MS with solution chemistry and laser ablation plays a suitable role as a micro-destructive technique, enabling trace element imaging and isotope ratio measurements at the trace and ultra-trace level in liquid and solid samples [18,19]. The laser ablation inductively coupled plasma-time of flight mass spectrometry (LA-ICP-TOF-MS) method has also been employed to determine trace species in rat brain tissues, vanilla samples and other materials. An important step is the preparation of standard samples for the detection of solid samples using LA-ICP-TOF-MS [20,21,22]. Sample preparation and introduction procedures are still necessary when employing these technologies, which is not suitable for the rapid measurement of trace elements in gas phase samples.

The rapid and precise element measurement of trace species with variable compositions is imperative in many applications. Laser diagnostics techniques such as laser-induced fluorescence (LIF), laser-induced breakdown spectroscopy (LIBS), tunable diode laser absorption spectroscopy (TDLAS), time-of-flight mass spectrometry (TOFMS) and so on [23,24] with their non-contact, fast response and multi-dimensional features have attracted a great deal of attention in various industries as qualitative and quantitative analytical detection techniques. LIBS is an analytical detection technique to measure the elemental composition based on atomic emission spectroscopy. LIBS has become a very popular analytical method in view of its unique features such as fast response, high sensitivity, real-time, non-contact and multi-element detection. The references about the fundamentals and applications of LIBS, which provide a systematic introduction including LIBS phenomena, LIBS principles, the process parameters, the instrumental components, the fascinating applications, *etc.* have been comprehensively reviewed [25,26]. The practical quantitative aspects and relevant applications, as well as the diagnostic aspects of laser-induced plasmas have been discussed and summarized in detail [27,28]. With the development of laser and detection systems, LIBS has shown a remarkably wide applicability in many fields, including combustion [29,30,31,32,33], metallurgy [34,35,36], food [37,38], human health [39,40] and others [41,42,43,44,45]. The number of applications is still growing. The advantages of the method are particularly significant in the areas of combustion, metallurgy and the study of harsh environments. One of the challenging targets of LIBS is the enhancement of the detection limits for gas phase materials. Though the experiments have been mainly applied for direct observation of post-breakdown processes, a new method to control the LIBS plasma generation process is necessary for the enhancement of detection limits, *i.e.*, low pressure and short pulse LIBS.

TOFMS with laser ionization processes is another appealing technique for the quantitative analysis of atomic and molecular ion signals with the features of increased sensitivity and rapid analysis. The detection limit of measured species using TOFMS is often ppb or less. TOFMS with several ionization processes has been applied to measure hydrocarbons and nanoparticle constituents at low concentration ranges of ppb–ppt [46,47,48,49,50], as well as waste disposal and treatment plants [51,52,53]. There are other developed TOFMS applications in several fields, such as the medical and pharmaceutical fields [54,55]. Considering the features of TOFMS for the detection of heavy atomic ions, fragmentation and signal intensity are the important factors for sensitive measurements. In order to eliminate or minimize the interference of fragmentation in the detection of heavy atoms, the method of laser breakdown time-of-flight mass spectrometry (LB-TOFMS) was employed to measure trace elements in gas phase samples. A laser beam is introduced to break and ionize the molecules and atoms in measured samples for the detection of atomic ion signals using TOFMS.

In this study trace species containing the elements mercury, iodine, strontium, cesium and arsenic were measured using low pressure LIBS and LB-TOFMS to verify their detection ability. Furthermore, low pressure LIBS and LB-TOFMS were utilized to measure mercury and iodine for a detailed comparison of the corresponding detection features.

## 2. Theory

**Figure 1:**
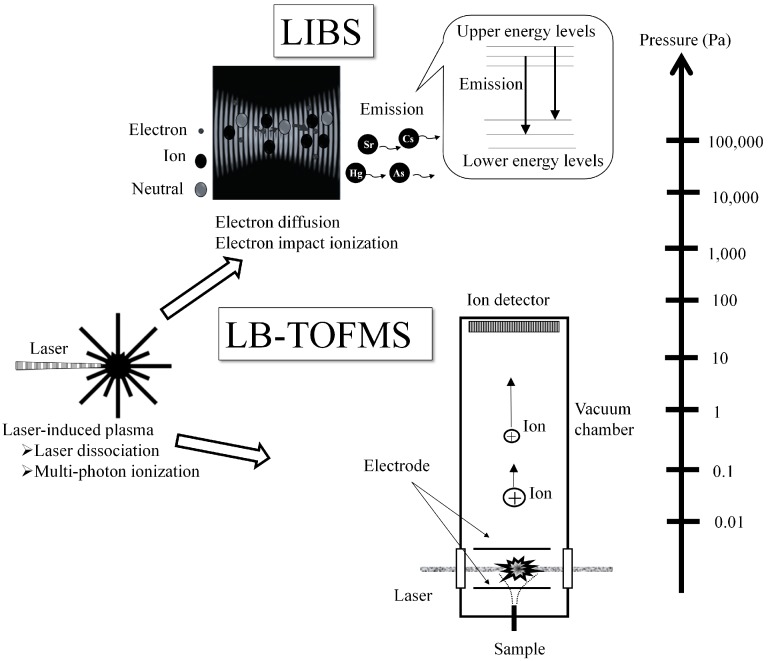
Different plasma generation processes and detection features of laser-induced breakdown spectroscopy (LIBS) and laser breakdown time-of-flight mass spectrometry (LB-TOFMS).

Figure 1 shows the plasma generation processes and detection features of LIBS and LB-TOFMS. Emission and ion signals are measured using the LIBS and LB-TOFMS detection systems respectively. The dominant phenomena in laser-induced plasma process differ depending on the pressure. At high pressure (atmospheric pressure), the electron diffusion and electron impact ionization processes are the major sources of the plasma generation. When the pressure is reduced, the effects of electron diffusion and electron impact ionization decrease compared with those under high pressure conditions [33]. The multi-photon ionization process becomes remarkable at low pressure, especially below 1 Pa. For example, in the case of LB-TOFMS, the plasma is mainly generated during the laser dissociation and multi-photon ionization processes, which contribute to the ion signal detection.

The creation and cooling processes of gas plasma in LIBS can be described as follows. The plasma core is firstly produced by the absorption of the incident laser energy, such as multi-photon ionization. In the multi-photon ionization process, a gas molecule or atom simultaneously absorbs a number of photons. When the absorbed energy is higher than its ionization potential, the gas molecule or atom is ionized. The creation of the plasma core induces the rapid growth of plasma through the absorption of the laser light by electrons and electron impact ionization processes in it. Electron impact ionization requires the existence of initial electrons generated by multi-photon ionization. The electrons then absorb more photons via the inverse bremsstrahlung process. The electrons with sufficient energy ionize other molecules by impact, leading to electron impact ionization and breakdown of the gas. After the termination of the laser pulse, the plasma continues to expand because of its high temperature and pressure gradients compared with the ambient condition. At the same time, recombination of electrons and ions proceeds due to the collision process and temperature decreases gradually compared with that in the plasma generation process. Therefore, the continuum emission is released by bremsstrahlung and recombination processes in the optically thin plasma. LIBS signals of ions, atoms and molecules arise in the plasma cooling period [24,56], which means the atomic and molecular emissions occur after a certain time delay. The delay time corresponding to the plasma temperature is an important parameter for LIBS measurements. There is an appropriate delay time—that is, the plasma temperature—to get a maximum signal to noise ratio. Generally, a shorter delay time is chosen in emissions at high pressure, and the longer delay time at low pressure. The continuum emission is considered as one of the major interferences with LIBS signals. Compared with LIBS, the laser breaks and ionizes the molecules and atoms in gas-phase samples at low pressure (usually less than 1 Pa) in the LB-TOFMS process and then the ion signals are detected using the TOFMS system. The sample is atomized and ionized through laser dissociation and multi-photon ionization processes. The electric field potential is simultaneously applied for acceleration of ions. The accelerated ions enter the drift region with no potential difference and undergo uniform motion. Differing from the emission detector of LIBS, an ion detector records the signals of ionized species and an ion counter takes over to digitize and display the results.

There are several interferences with target signals in LIBS, generally including the continuum emission from plasma itself, coexisting molecular and atomic emissions, noise from detectors, *etc.* At high pressure, the main interference is the continuum emission from the plasma itself, the intensity of which is proportional to the biquadratic temperature. The generated plasma is dense and the electrons, ions and neutral species collide frequently with each other. The temperatures of electrons, ions and neutrals become approximately the same through the violent collisions to transfer energy in high pressure plasma, generally called thermal plasma. At low pressure, however, the density of plasma is rather low and the few opportunities for collisions between electrons and other particles cause a large difference of each particle’s kinetic energy. Once the initial electrons and ions are produced, they are accelerated by absorbing laser energy intensely. Due to the mass difference of charged particles, the kinetic energy of electron is much higher. Since the neutral species obtain their energy by particle (electron, ion and neutral) collisions, the kinetic energy of neutrals is lower compared with that of the charged particles. These particles in plasma are not under local thermodynamic equilibrium (LTE) conditions. When *T_e_*, *T_i_* and *T_n_* are defined as the kinetic temperatures of electrons, ions and neutrals respectively, the temperatures are in the order *T_e_* >> *T_i_*, *T_e_* >> *T_n_*, which is called cold plasma. In this case, the departure from LTE produces a population of excited levels differing from the Boltzmann distribution. Populations of ions and neutrals in the upper energy levels are larger than that under LTE conditions [57]. This phenomenon becomes eminent under low pressure conditions. The collisions reduce and plasma expansion becomes faster. The interferences from coexisting molecular and atomic emissions appear from the products of plasma generation process, where the temperature of each atom also shows a different value. Compared with this interference, the influence of the continuum emission from the plasma itself is insignificant at low pressure and some of the atomic emissions become eminent, which means the enhancement of the detection ability of this specific atom. On the other hand, laser breakdown TOFMS was employed with direct introduction of gas-phase samples into the vacuum chamber for the detection of trace species in gases. The pressure in LB-TOFMS is usually less than 1 Pa. The molecules and atoms are broken and transferred to their ionization level under the reduced pressure conditions [50] where the electric field potential is simultaneously applied for acceleration of the ions. TOFMS distinguishes the ions of different atoms or molecules based on their arrival time to an ion detector. These interferences usually restrict the sensitivity. Because of the low pressure condition, the electron impact ionization process becomes less important in LB-TOFMS compared with that in LIBS. The major interference in LB-TOFMS is the partial fragmentation and daughter ions at the mass region of the target atomic ion. The fragmentation and signal intensity are the important factors for sensitive LB-TOFMS measurements.

The emission and ion signals are affected by the laser power, pressure, wavelength, and pulse width in each plasma generation process. The influencing parameters in LIBS and LB-TOFMS processes are not completely consistent because of the different detection items, such as delay time in LIBS. The influencing parameters and results of two detection systems are summarized in Table 1. The electron impact ionization process is significant at high pressure (atmospheric pressure) because the electron–atom or electron–ion collisions have sufficient opportunities to occur. The LIBS plasma is mainly induced by electron impact ionization at high pressure, which leads to the high plasma temperature and the increased continuum emission [58]. When reducing the pressure, this influence will decrease. However, the electron impact ionization process is essential to the generation of LIBS plasma. In other words, there is the lower pressure limit in LIBS. Because the pressure in TOFMS is less than 1 Pa, the particle feature of generated plasma is utilized for ion detection. Within such a low pressure range, the ion number density increases with the increase of the pressure until the upper pressure limit of TOFMS working conditions.

**Table 1:** Influence of LIBS and LB-TOFMS detection systems. The key physical processes consist of (1) multi-photon ionization, (2) electron impact ionization and (3) recombination.

Parameter	Significant Influence			
	LIBS		LB-TOFMS	
	Physical	Effect	Physical	Effect
Pressure	(2)	Plasma temperature	---	Ion number density
		continuum emission		
Laser power	(2)	Plasma temperature	(3)	Ion number density
		Ionization and excitation		
Delay time	---	Plasma temperature	---	---
Wavelength	(1)	Ionization and excitation	(1)	Ionization
Pulse width	(1), (2)	Ionization and excitation	(1)	Ionization

In laser-induced plasma processes, more electron and ion particles can be produced when the laser power is increased. Due to the collision of different particles, the plasma temperature will increase and the recombination of ions and electrons will occur. Usually, the upper level energies of the trace species, such as mercury, iodine, strontium and so on, are low, and they can be ionized and excited at low laser power leading to larger ionization and excitation. In the LIBS process, the emission signal occurs during the plasma cooling process, that is, after a certain time delay. Because of the different photon energy at different laser wavelengths, the multi-photon ionization process can be influenced by irradiated laser wavelength in LIBS and LB-TOFMS. The laser-induced plasma process, especially the electron impact ionization process, can be controlled using different pulse width lasers, which can also affect the multi-photon ionization process. Short wavelength and pulse irradiation with its own pulse energy and pulse width allow for a specific and larger ionization and excitation that could yield emission and ion signals more efficiently. In order to clarify these influences, several samples were measured using LIBS and LB-TOFMS in this study.

## 3. Experiment

**Figure 2:**
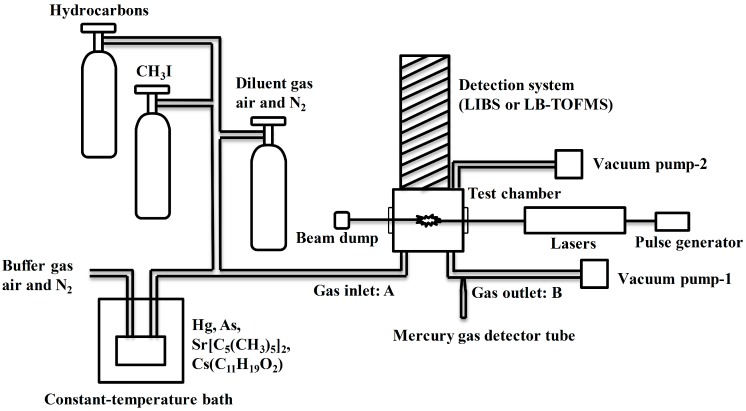
Schematic diagram of the experimental system.

**Figure 3:**
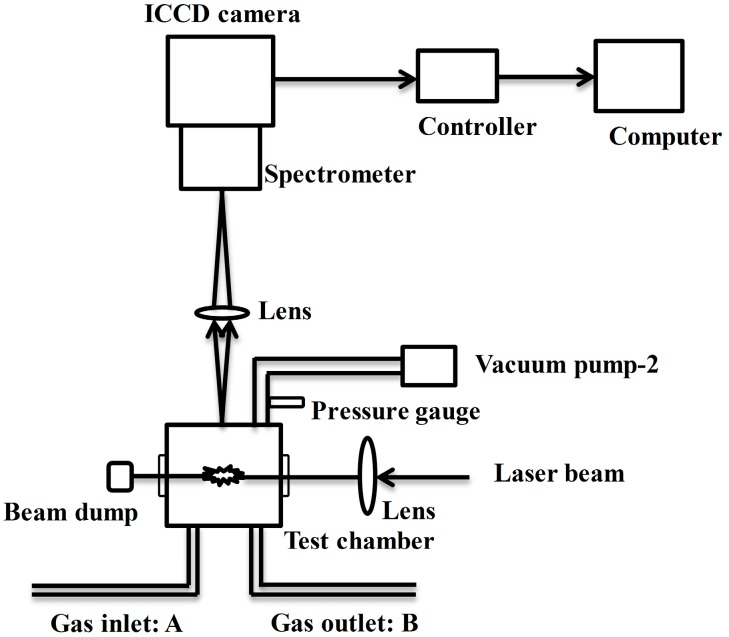
Schematic diagram of the laser-induced breakdown spectroscopy (LIBS) system.

**Figure 4:**
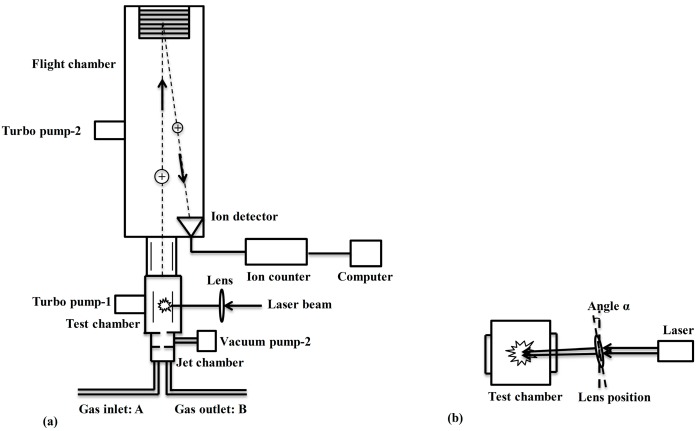
Schematic diagram of the laser breakdown time-of-flight mass spectrometry (LB-TOFMS) system. (**a**) Detection system of laser breakdown time-of-flight mass spectrometry (LB-TOFMS); (**b**) Different position of the focal lens in the LB-TOFMS system.

The schematic diagrams of the experimental system in this study are illustrated in Figure 2, Figure 3 and Figure 4. The overall setup including input and detection systems (LIBS and LB-TOFMS) is shown in Figure 2. Hg, CH_3_I (Taiyo Nippon Sanso, Tokyo, Japan), As, Sr[C_5_(CH_3_)_5_]_2_ (Kojundo Chemical Laboratory Co., Saitama, Japan), and Cs(C_11_H_19_O_2_) (Cs dpm, Kojundo Chemical Laboratory Co. Saitama, Japan) were employed to evaluate the elemental detection features of mercury, iodine, arsenic, strontium and cesium using LIBS and LB-TOFMS. Here, Hg and CH_3_I were mainly employed for comparison of two detection systems. Hydrocarbons, the standard CH_3_I gas in N_2_ with concentration of 101 ppm and the gaseous mixtures of Hg, As, Sr[C_5_(CH_3_)_5_]_2_ and Cs(C_11_H_19_O_2_) from the constant-temperature bath according to the vaporizing pressure (VP) were introduced with buffer gases of air and N_2_ into the measurement chambers. The temperatures of Hg, As, Sr[C_5_(CH_3_)_5_]_2_ and Cs(C_11_H_19_O_2_) in the constant-temperature bath were 370 K (VP: 39 Pa), 490 K, 420 K (VP: 2.9 Pa) and 470 K, respectively. Air and N_2_ were also used as diluent gases to reduce the concentration of samples introduced to the measurement chambers. Mercury concentration was determined using a mercury gas detector tube (K.K. No. 142S Komyo Rikagaku Kogyo, Kawasaki, Japan) at the gas outlet. Because gas phase samples were employed in this study, molar fraction units, such as ppm or ppb, was used to specify the concentration. The concentration in the unit of mass per volume can be calculated by the atomic mass of the target species. For example, mercury concentration of 1 ppb is 0.0089 mg/m^3^(NTP) and iodine concentration of 1 ppb is 0.0056 mg/m^3^(NTP).

Different pulse width lasers of nanosecond, 150 ps and 35 ps were employed for LIBS and LB-TOFMS. A nanosecond laser 1 is a pulsed Nd:YAG laser (LOTIS TII, Minsk, Belarus, LS-2134UTF, 5–8 ns, 10 Hz, beam diameter: 6 mm) operated at fundamental wavelength of 1064 nm and fourth harmonic wavelength of 266 nm. A nanosecond laser 2 employed is also a pulsed Nd:YAG laser (Quanta-Ray, Santa Clara, CA, USA, Pro-230, 6–12 ns, 10 Hz, beam diameter: 9 mm) operated at fundamental wavelength of 1064 nm and second harmonic wavelength of 532 nm. The nanosecond laser 3 (Quantel, Les Ulis, France, Brilliant b, 6 ns, 10 Hz, beam diameter: 9 mm) was operated at fundamental wavelength of 1064 nm. The output wavelength of picosecond laser 1 (EKSPLA, Vilnius, Lithuania, SL312, 150 ps, 10 Hz, beam diameter: 10 mm) was fundamental wavelength of 1064 nm. The picosecond laser 2 (Quantel, Les Ulis, France, YG901C-10, 35 ps, 10 Hz, beam diameter: 9.5 mm) was operated at fundamental wavelength of 1064 nm and second harmonic wavelength of 532 nm. The used lasers and the specific have been summarized in Table 2. The power of the lasers employed in this study was measured as an average value (energy per pulse). The output laser beam was focused into the measurement chambers.

**Table 2:** Nd:YAG lasers and the specific.

Nd:YAG Laser	Pulse Width@1064 nm	Wavelength (nm)	LB-TOFMS	LIBS
LOTIS TII, LS-2134UTF	5–8 ns	266, 1064	HC	---
Quanta-Ray, Pro-230	6–12 ns	532, 1064	HC, Hg, I, As	Sr, Cs, I
Quantel, Brilliant b	6 ns	1064	Hg, I	Hg, I
EKSPLA, SL312	150 ps	1064	---	Hg, I
Quantel, YG901C-10	35 ps	532, 1064	Hg, I	Hg, I

The experimental apparatus of laser-induced breakdown spectroscopy (LIBS) fundamentally consisted of vacuum chamber, lenses, spectrometer, ICCD camera and auxiliary equipment, as shown in Figure 3. The output laser beam was focused into the measurement chamber using a lens with a focal length of 80 mm. The measurement chamber designed for this experiment was a vacuum cell with four quartz windows and its internal volume was about 200 cm^3^. The length from the center of the chamber to the quartz windows was 65 mm. Perpendicular to the laser propagation direction, emission from another window of the chamber was focused onto the spectrometer slit. The focal length of the lens between the vacuum chamber and the spectrometer was 60 mm. Emission signals were finally detected by combination of a spectrometer (CT-10S, JASCO, Tokyo, Japan), an ICCD camera (Model ITEA/CCD-576-S/RB-E Princeton Instruments Inc., Princeton, NJ, USA) and auxiliary equipment. The row of the ICCD camera was used as wavelength axis. Each spectrum was evaluated by summing the emission intensity of each column. The pressure was measured using a pressure gauge (SE1000-SNV-420T1, Tem-Tech Lab, Tokyo, Japan) installed at the evacuation port of the chamber. The range of pressure was around 400 Pa–10 kPa for low pressure LIBS. In the measurement of iodine, the iodine signal was located at a vacuum UV region such as 183 nm, in which there was the absorption of O_2_. Therefore pure N_2_ was purged along the detection path from the vacuum chamber to the spectrometer.

Figure 4 shows the experimental apparatus for LB-TOFMS. It consisted of the jet chamber, test chamber, flight chamber (D-850 AREF; drift tube length: 500 mm, R.M. Jordan Co., Grass Valley, NV, USA), ion detector (R.M. Jordan Co., Grass Valley, NV, USA, 40 mm MCP Z-gap detector), ion counter (Model SR430 multi-channel scaler, SRS, Sunnyvale, CA, USA) and auxiliary equipment. The jet chamber was employed to ensure heavy atoms go straight ahead to the test chamber for the measurement of heavy atoms. The laser beam was focused into the test chamber. Because of the recombination of ions with electrons, the focal area was enlarged by tilting the focal lens to improve the detection ability of this method, as shown in Figure 4b. Angle α was defined to represent different position of the focal lens [59]. The temperatures of gas inlet and outlet pipes were controlled at 423 K. There are two turbo pumps (MVP 055-3, Pfeiffer Vacuum, Asslar, Germany) belonging to the test chamber and the flight chamber respectively. The pressure in the test chamber was employed as a pressure indicator under different experimental conditions. The pressure was less than 1 Pa for LB-TOFMS. The data were accumulated from 50 laser shots (5 s) to 1200 laser shots (120 s) and transferred to the computer for analyses [33,59].

## 4. Results and Discussion

### 4.1. Laser Breakdown Process and Detection Features 

Mercury, iodine, arsenic, strontium and cesium were measured by the LIBS and LB-TOFMS systems employing samples of Hg, CH_3_I, As, Sr[C_5_(CH_3_)_5_]_2_ and Cs(C_11_H_19_O_2_). In this paper, Hg and CH_3_I were mainly employed for comparison of the detection features of the LIBS and LB-TOFMS systems. The samples were broken by the laser irradiation focused into the measurement chambers in the LIBS and LB-TOFMS systems. After the laser dissociation process, the plasma was produced through the multi-photon ionization and electron impact ionization processes. The emission and ion signals were detected using the detectors in each system, respectively. Table 3 lists the specific wavelengths and upper level energy of the measured species in this study.

**Table 3:** Upper level energy of measured species.

Species	Wavelength (nm)	Upper Level Energy (cm^−1^)
Hg(I)	253.7	39,412.2 [60]
NO	258	38,759.7 [61]
I(I)	178.3	56,092.88 [62]
I(I)	179.9	63,186.76 [62]
I(I)	183	54,633.5 [62]
I(I)	206.2	56,092.88 [62]
N(I)	174.3	86,220.5 [63]
C(I)	193.1	61,981.82 [64]
Sr(I)	460.7	21,698.45 [65]
Cs(I)	852.1	11,732.31 [66]

**Figure 5:**
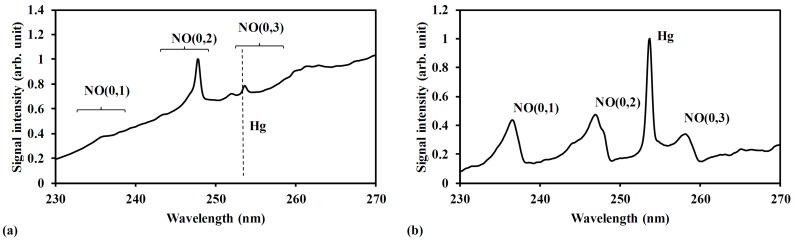
LIBS spectra of Hg in air at different pressure. (**a**) Pressure 30 kPa. Conditions: ns 1064 nm laser irradiation, delay time 2060 ns, power 600 mJ/p, beam diameter 9 mm, gate width 10 μs; (**b**) Pressure 2000 Pa. Conditions: ns 1064 nm laser irradiation, delay time 560 ns, power 800 mJ/p, beam diameter 9 mm, gate width 30 μs. The signal was normalized by the maximum signal intensity in each measurement result.

Figure 5 shows the emission signals of Hg in air detected by LIBS under high pressure and low pressure conditions. Comparing the measurement results at different pressure, the Hg(I) line at 253.7 nm was distinguished obviously with the high signal intensity at low pressure. The interference of the continuum emission from plasma itself reduced dramatically under low pressure condition, as shown in Figure 5b, which illustrates how the interference from coexisting molecular emissions became the main influencing factor. Hg signal with sufficient signal to noise ratio was not able to achieve by the optimization of the delay time and the gate width at 30 kPa [67]. The NO emissions formed during the plasma generation and cooling process of N_2_ and O_2_ in the air became the interference of coexisting molecular emissions [68,69]. The (0, 3) emission band of the A^2^Σ → X^2^П electronic transition of NO centered at 258 nm was the main interference with Hg signal. In Figure 5b the gate width was 30 μs at low pressure, which was longer than that of 10 μs at high pressure. This can be explained by the lifetime of plasma and the atomic emission features at different pressure.

**Figure 6:**
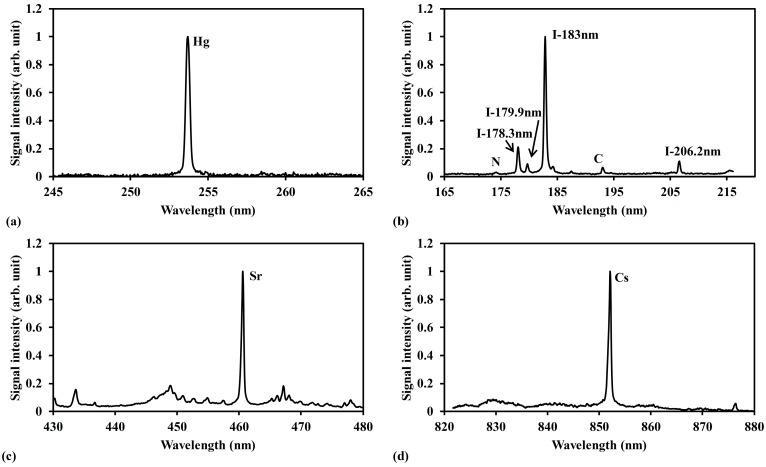
LIBS spectra of Hg, CH_3_I, Sr[C_5_(CH_3_)_5_]_2_ and Cs(C_11_H_19_O_2_) in N_2_. (**a**) Mercury measurement result. Conditions: ns 1064 nm laser irradiation, pressure 6600 Pa, delay time 1060 ns, power 400 mJ/p, beam diameter 9 mm, gate width 30 μs. (**b**) Iodine measurement result. Conditions: ns 1064 nm laser irradiation, pressure 400 Pa, delay time 1060 ns, power 400 mJ/p, beam diameter 9 mm, gate width 30 μs. (**c**) Strontium measurement result. Conditions: ns 1064 nm laser irradiation, pressure 2600 Pa, delay time 10 μs, power 1000 mJ/p, beam diameter 9 mm, gate width 30 μs. (**d**) Cesium measurement result. Conditions: ns 1064 nm laser irradiation, pressure 10 kPa, delay time 100 μs, power 1000 mJ/p, beam diameter 9 mm, gate width 300 μs. The signal was normalized by the maximum signal intensity in each measurement result.

Figure 6 shows the measurement results of Hg, CH_3_I, Sr[C_5_(CH_3_)_5_]_2_ and Cs(C_11_H_19_O_2_) in buffer gas of N_2_ at low pressure. The apparent signals of Hg(I) line at 253.7 nm, I(I) line at 183 nm, Sr(I) line at 460.7 nm and Cs(I) line at 852.1 nm can be detected with little interference of continuum emission and coexisting materials. There were several detectable iodine signals in Figure 6b. The iodine emission of I(I) line at 183 nm was the representative signal in this study. At low pressure, the plasma temperature becomes low due to the diminished influence of the electron impact ionization process, resulting in the decrease of interference of continuum emission from plasma itself. Low pressure LIBS (400 Pa–10 kPa), therefore, has been applied to measure the trace species of mercury and iodine for exhaustive comparison of the detection features of the LIBS and LB-TOFMS systems.

In the LB-TOFMS detection system, because of the low pressure in the chambers (usually less than 1 Pa), laser dissociation and multi-photon ionization processes become dominant to ionize the atoms or molecules. The major interferences of LB-TOFMS are the partial fragmentation and daughter ions from the materials with target atomic ion signals. In order to detect the elemental compositions in high-mass region compounded by complex molecules, such as hydrocarbons and particulates, LB-TOFMS was applied to the mixture of hydrocarbons (mixture of *p*-C_7_H_6_Cl_2_, C_7_H_8_, C_6_H_5_C_2_H_3_, *p*-C_8_H_10_, *p*-C_6_H_4_(C_2_H_5_)_2_ and C_6_H_3_(CH_3_)_3_) using 266 nm and 1064 nm laser irradiation [59]. The measurement result using 266 nm laser irradiation shows the serious interference of the partial fragmentation. However, the result of 1064 nm laser irradiation is very distinct without any interference in the *m/z* 30–300 mass region due to the suitable breakdown. The laser operated at 532 nm and 1064 nm was also employed to measure the hydrocarbon *p*-C_8_H_10_. The *m/z* 30–300 mass region is very clear in both cases [59]. Accordingly the trace heavy atoms of As, Sr, I, Cs and Hg can be distinguished without the interference of partial fragmentation using 1064 nm and 532 nm laser irradiation.

**Figure 7:**
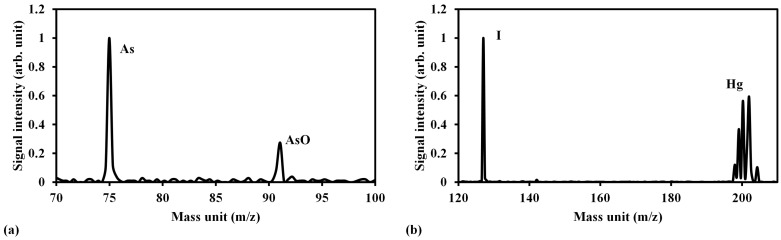
LB-TOFMS mass spectra of Hg, CH_3_I and As in N_2_ using 532 nm laser irradiation. (**a**) Arsenic measurement result. Conditions: buffer gas N_2_, pressure 0.5 Pa, power 100 mJ/p, lens angle α 0°; (**b**) Mercury and iodine measurement result. Conditions: buffer gas N_2_, pressure 0.15 Pa, power 100 mJ/p, lens angle α 10°, mercury concentration 3.2 ppm, iodine concentration 4.8 ppm. The signal was normalized by the maximum signal intensity in each measurement result.

Figure 7 shows the LB-TOFMS mass spectra of mercury, iodine and arsenic in buffer gas of N_2_ using 532 nm laser irradiation. It is recognized that the mass spectra of Hg^+^, I^+^ and As^+^ without any interference were detected. Because of the high resolution of TOFMS, the isotopes can be recognized precisely, which is one of the different detection features compared with LIBS, as illustrated in Figure 7b. According to these measurement results, the trace species of mercury and iodine were measured using the low pressure LIBS and LB-TOFMS systems with 1064 nm and 532 nm laser irradiation under various experimental conditions in this study. The comprehensive discussion will be clarified as follows.

### 4.2. Pressure Effect on Detection Ability

**Figure 8:**
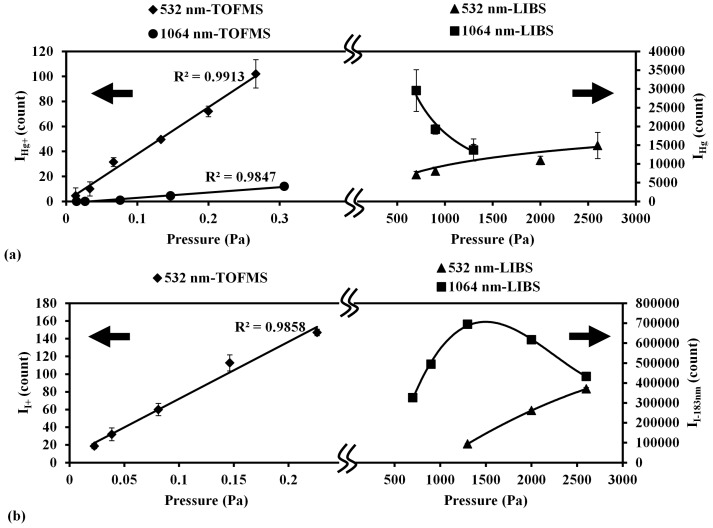
Pressure dependence of ion and emission signal intensities using 532 nm and 1064 nm laser irradiation in the LB-TOFMS and LIBS systems. (**a**) Mercury ion and emission signal intensities. Conditions: LB-TOFMS, ns 1064 nm laser irradiation, buffer gas N_2_, power 838 mJ/p, mercury concentration 8.3 ppm, lens angle α 0°. ns 532 nm laser irradiation, buffer gas N_2_, power 100 mJ/p, mercury concentration 0.86 ppm, lens angle α 10°. LIBS, ns 532 nm and 1064 nm laser irradiation, delay time 1060 ns, power 400 mJ/p, beam diameter 9 mm, gate width 30 μs; (**b**) Iodine ion and emission signal intensities. Conditions: LB-TOFMS, ns 532 nm laser irradiation, buffer gas N_2_, power 40 mJ/p, iodine concentration 5.05 ppm, lens angle α 10°. LIBS, ns 532 nm and 1064 nm laser irradiation, delay time 1060 ns, power 400 mJ/p, beam diameter 9 mm, gate width 30 μs.

Mercury and iodine were measured using the low pressure LIBS and LB-TOFMS systems with 1064 nm and 532 nm laser irradiation at different pressures, respectively. In order to facilitate comparison of the detection features of the LIBS and LB-TOFMS systems, the measurement results using LIBS and LB-TOFMS were merged. Figure 8 shows the pressure dependence of mercury and iodine signal intensities using the LB-TOFMS and LIBS systems.

The intensities of the Hg^+^ signal (I_Hg+_) and I^+^ signal (I_I+_) show a linear increase with the increase of pressure in the LB-TOFMS system. Within such a low pressure range (less than 1 Pa), the ion number density increased when the pressure increased because of the non-collisional feature in TOFMS. In the case of LIBS, the tendencies of Hg emission signal (I_Hg_) and iodine emission signal (I_I-183nm_) between 1064 nm and 532 nm are different under each condition. There is the delay time effect in LIBS process and this effect shows different tendency in 1064 nm and 532 nm laser irradiation and strong dependence on pressure. In LIBS measurement of mercury in air using 1064 nm laser irradiation at higher pressure, a reasonable Hg emission signal could not be detected under the same experimental conditions. However, in the case of iodine measurement using 1064 nm laser irradiation, the intensity of I-183 nm emission shows different tendency due to different upper level energies of Hg(I) line at 253.7 nm and I(I) line at 183 nm, as listed in Table 3.

In the LB-TOFMS process, the plasma is mainly produced by laser dissociation and multi-photon ionization processes. Comparing I_Hg+_ between 532 nm and 1064 nm laser irradiation shown in Figure 8a, the signal intensity was higher in the case of 532 nm laser irradiation because of the higher photon energy concerning the multi-photon ionization process compared with that of 1064 nm laser irradiation [70]. In the LIBS process, the plasma is mainly produced by the multi-photon ionization and electron impact ionization processes. The emission signals arise in the plasma cooling period after plasma growth by the electron impact ionization process that can be affected by laser wavelength and laser power. I_Hg_ was higher when using 1064 nm laser irradiation. Taking into account the comparison of Hg^+^ results using different wavelengths, I^+^ was measured using 532 nm laser irradiation at different pressure. Figure 8b shows the pressure dependence of I^+^ signal intensity (I_I+_) using 532 nm laser irradiation, which is linear in accordance with the results of Hg^+^. In the case of 1064 nm laser irradiation a reasonable I^+^ signal was undetectable under the same laser irradiation conditions. In LIBS measurement results, the intensity of iodine emission signal (I_I-183nm_) was also higher when using 1064 nm laser irradiation, which shows the consistent results for the mercury measurement.

**Figure 9:**
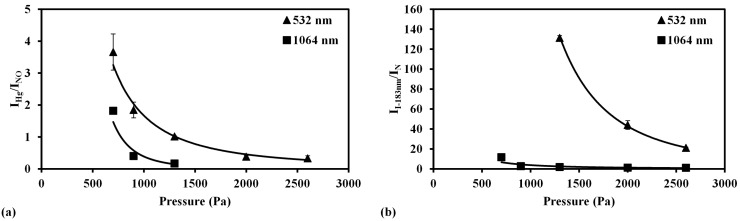
Pressure dependence of signal to noise ratio using 532 nm and 1064 nm laser irradiation in LIBS. (**a**) The ratio of I_Hg_/I_NO_. Conditions: ns 532 nm and 1064 nm laser irradiation, delay time 1060 ns, power 400 mJ/p, beam diameter 9 mm, gate width 30 μs; (**b**) The ratio of I_I-183nm_/I_N_. Conditions: ns 532 nm and 1064 nm laser irradiation, delay time 1060 ns, power 400 mJ/p, beam diameter 9 mm, gate width 30 μs.

In LIBS, the signal to noise ratio was utilized to evaluate the detection ability. The coexisting molecular and atomic emissions became the main interference under low pressure conditions. The coexisting molecular and atomic emissions in the mercury measurement in air are the NO emissions due to the recombination of N and O in the plasma generation process [69]. In the measurement of iodine, the buffer gas was N_2_ that produced N atom emissions which appear around the iodine emission wavelength region. N emission at 174.3 nm was determined as the coexisting atomic emission to clarify the plasma generation process including the multi-photon ionization and electron impact ionization processes. The intensity ratios of Hg emission to NO emission (I_Hg_/I_NO_) and I-183nm emission to N emission (I_I-183nm_/I_N_) were defined to evaluate the emission features of mercury and iodine compared with the emissions of NO centered at 258 nm and N at 174.3 nm from buffer gases. Figure 9 shows the pressure dependence of I_Hg_/I_NO_ and I_I-183nm_/I_N_ ratios using the LIBS system. When the pressure decreased the ratios of I_Hg_/I_NO_ and I_I-183nm_/I_N_ increased, which indicates the influence of the coexisting materials (air and N_2_) was reduced to enhance the detection ability. The comparison between 532 nm and 1064 nm laser irradiation at different pressure reveals the higher intensity ratios of I_Hg_/I_NO_ and I_I-183nm_/I_N_ in the case of 532 nm laser irradiation because of the larger ionization and excitation of mercury and iodine.

In the plasma generation process, when the pressure is reduced, the density of ions and electrons in the plasma becomes lower and the effect of electron impact ionization, which creates lots of N and O ions, is reduced [56]. Therefore, when the pressure decreased, the ratios of I_Hg_/I_NO_ and I_I-183nm_/I_N_ increased. This can also be recognized from the comparison of ion signals, such as N_2_^+^, N^+^, I^+^ and Hg^+^. Hg^+^ and I^+^ were mainly produced by the multi-photon ionization process, which can be explained according to the LB-TOFMS measurement results [59]. The concentrations of Hg and CH_3_I in buffer gas of N_2_ were at the ppm level. However, the signal intensities of Hg^+^ and I^+^ compared with that of N^+^ and N_2_^+^ were not directly proportional to the concentrations. Laser dissociation and multi-photon ionization are the dominant processes for plasma generation of Hg^+^ and I^+^ signals in LB-TOFMS. Considering this fact, in LIBS process, mercury and iodine emission signals were also mainly generated in the multi-photon ionization process. NO and N signals were enhanced intensely by the electron impact ionization process, which becomes less dominant when the pressure is reduced, resulting in the increase of the ratios of I_Hg_/I_NO_ and I_I-183nm_/I_N_. According to the results of low pressure LIBS and LB-TOFMS, the detection ability of trace species can be enhanced under low pressure conditions due to the control of the electron impact ionization process.

### 4.3. Laser Power Effect on Detection Ability

**Figure 10:**
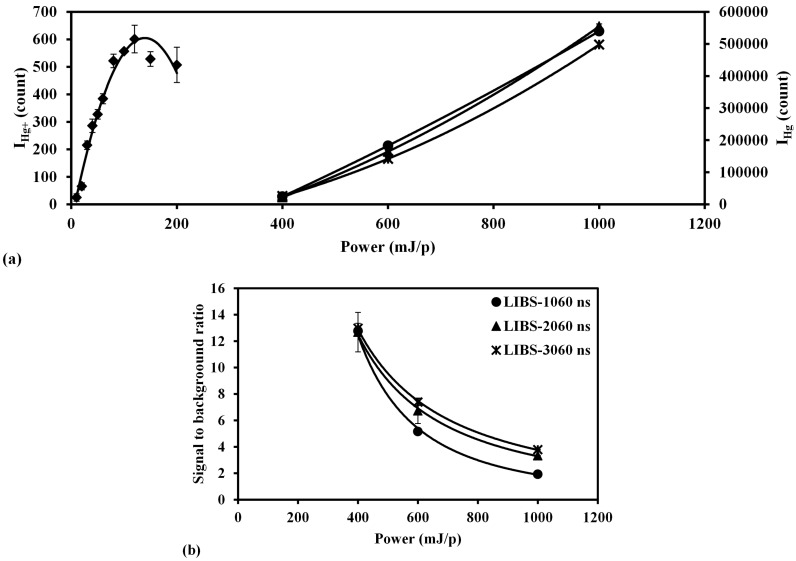
Dependence of Hg+ and Hg emission signals on laser power using LB-TOFMS and LIBS. (**a**) Hg^+^ and Hg emission signal intensities; (**b**) Hg signal to background ratio in LIBS. Conditions: LB-TOFMS, ns 532 nm laser irradiation, buffer gas N_2_, pressure 0.15 Pa, mercury concentration 3.2 ppm, lens angle α 10°. LIBS, ns 1064 nm laser irradiation, buffer gas N_2_, pressure 2600 Pa, beam diameter 9 mm, gate width 30 μs, delay time 1060 ns, 2060 ns and 3060 ns.

The laser power influence of LIBS and LB-TOFMS has been studied by the measurement of trace mercury species under different laser power conditions. The measurement results of LB-TOFMS using 532 nm laser irradiation showed enhanced signals [59]. In the case of LIBS measurement, mercury was also measured under various experimental conditions including wavelength, pulse width and delay time [33]. The reasonable and comparable results can be acquired using 1064 nm laser irradiation under these conditions. Figure 10 shows the laser power dependence of ion and emission signals in LB-TOFMS and LIBS, as well as Hg signal to background ratio in LIBS.

The variation of I_Hg+_ based on the laser power in LB-TOFMS is presented in Figure 10a, which shows that I_Hg+_ increased first and then decreased when the laser power was increased. The ion signal intensity increases as the laser power increases according to the multi-photon ionization process. However, under the experimental condition, the laser power exceeding 120 mJ/p caused the reduction of Hg^+^ signal intensity. This phenomenon may have occurred due to the recombination of ions with electrons under high laser power conditions. As the laser power increased, more N_2_ molecules were broken and ionized to produce dense ions and electrons. Because of the high recombination rate of Hg^+^ with electrons [59], I_Hg+_ decreased in high laser power conditions. Therefore the focal area was enlarged by tilting the focal lens to reduce the recombination effect, as Figure 4b shows. Trace mercury species were also measured using the LIBS system under different laser power conditions. Figure 10a also shows the laser power dependence of Hg emission signal using 1064 nm laser irradiation for different delay times. The Hg emission signal intensity increased when the laser power increased due to the main effect of electron impact ionization process.

The laser power dependence of Hg emission signal was measured using 1064 nm laser irradiation in N_2_ at a pressure of 2600 Pa. The background noise around 253.7 nm was employed to evaluate the detection ability using the Hg signal to background ratio. The signal to background ratio at low laser power of 400 mJ/p was improved compared with that at high laser power of 1000 mJ/p, as shown in Figure 10b. When the laser power increased, the intensities of all the emission signals increased, especially the interference emissions due to the effect of electron impact ionization process, resulting in a decrease of the signal to background ratio. An identical tendency was observed in the iodine measurement using the LB-TOFMS and LIBS systems.

In the LIBS and LB-TOFMS systems, when the laser power increased the emission and ion signal intensities increased too. After a certain laser power, more electrons can be produced to enhance the emission signals, especially the interference emissions for LIBS and the recombination rate of ions with electrons for LB-TOFMS, leading to a decline in the detection ability. Therefore it is necessary to determine the suitable laser power for these measurements to enhance the detection ability considering the breakdown threshold and plasma growth.

### 4.4. Influence of Delay Time on Emission Signals

**Figure 11:**
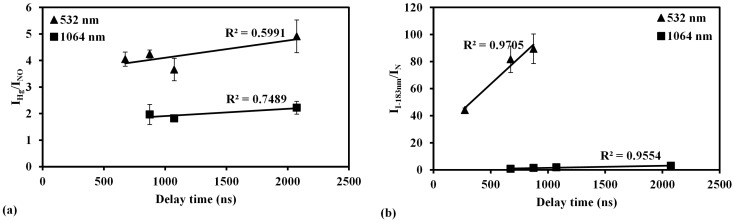
Comparison of I_Hg_/I_NO_ and I_I-__183nm_/I_N_ dependence on delay time between 1064 nm and 532 nm laser irradiation using LIBS. (**a**) The ratio of I_Hg_/I_NO_. Conditions: ns 532 nm and 1064 nm laser irradiation, power 400 mJ/p, beam diameter 9 mm, pressure 700 Pa, gate width 30 μs; (**b**) The ratio of I_I-183nm_/I_N_. Conditions: ns 532 nm and 1064 nm laser irradiation, power 400 mJ/p, beam diameter 9 mm, pressure 1300 Pa, gate width 30 μs.

LIBS signals appear during the plasma cooling process, which means the atomic and molecular emissions arise after a certain time delay. Therefore, the delay time corresponding to the plasma temperature is an important parameter for LIBS measurement. Comparing the influencing parameters of LIBS and LB-TOFMS, the delay time is not the basic parameter for LB-TOFMS due to different features of plasma and detection signals. There is an appropriate delay time—that is, the plasma temperature—to get a maximum signal to noise ratio in LIBS. The influence of delay time on emission signals was discussed when describing the mercury and iodine measurement results, which is beneficial to understand the plasma generation process in LIBS. The delay time dependence of I_Hg_/I_NO_ and I_I-183nm_/I_N_ was measured using 1064 nm and 532 nm laser irradiation respectively, as shown in Figure 11. The ratios of I_Hg_/I_NO_ and I_I-183nm_/I_N_ were improved using 532 nm laser irradiation due to larger ionization and excitation of mercury and iodine signals in multi-photon ionization process, which was consistent with the measurement results of the pressure dependence using 1064 nm and 532 nm laser irradiation.

At low pressure, the delay time was not a determining factor for I_Hg_/I_NO_ in mercury measurement because of their similar upper level energies, as shown in Figure 11a. In the case of iodine measurement using 532 nm laser irradiation shown in Figure 11b, I_I-183nm_/I_N_ increased when the delay time increased. The temperature of plasma reduced as increasing the delay time [58], leading to the decrease of coexisting N atom emission due to the higher upper level energy of N(I) line at 174.3 nm compared with that of I(I) line at 183 nm, as listed in Table 3. Therefore the intensity ratio of I_I-183nm_/I_N_ increased.

### 4.5. Pulse width Effect on Detection Ability

**Figure 12:**
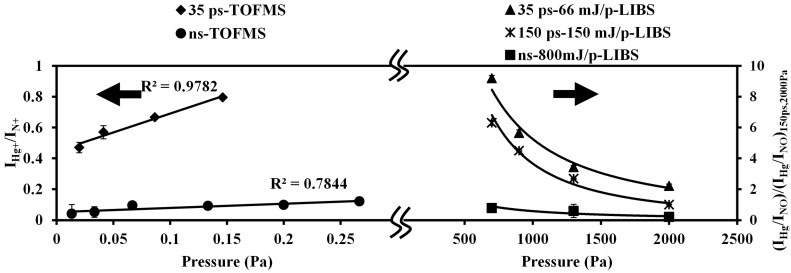
Pulse width influence of I_Hg+_/I_N+_ and I_Hg_/I_NO_ using LB-TOFMS and LIBS at different pressure. Conditions: LB-TOFMS, 35 ps 532 nm laser irradiation, buffer gas N_2_, power 28 mJ/p, mercury concentration 1 ppm, lens angle α 20°. ns 532 nm laser irradiation, buffer gas N_2_, power 100 mJ/p, mercury concentration 0.86 ppm, lens angle α 10°. LIBS, delay time 1060 ns, 1064 nm, beam diameter 9 mm (ns laser), beam diameter 10 mm (150 ps laser), beam diameter 9.5 mm (35 ps laser), gate width 30 μs. The values of I_Hg_/I_NO_ were all referred to that obtained using 150 ps laser irradiation at 2000 Pa.

**Figure 13:**
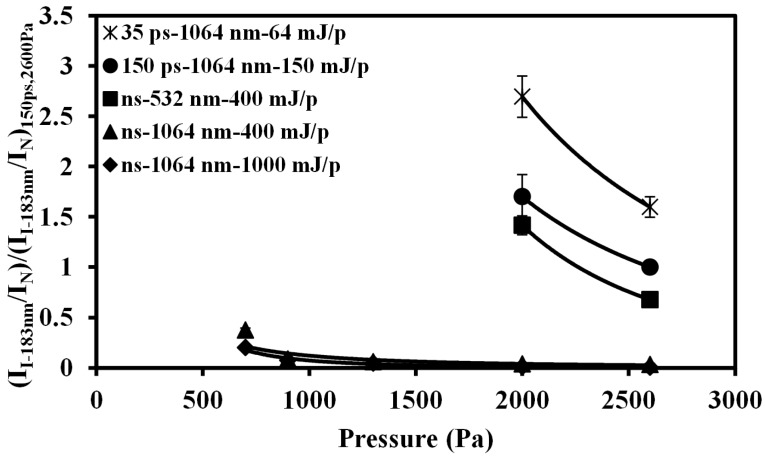
Pressure dependence of I_I-__183nm_/I_N_ using LIBS under various conditions. Conditions: delay time 1060 ns, beam diameter 9 mm (ns laser), beam diameter 10 mm (150 ps laser), beam diameter 9.5 mm (35 ps laser), gate width 30 μs. The values of I_I-183nm_/I_N_ were all referred to that obtained using 150 ps laser irradiation at 2600 Pa.

Different pulse width lasers, such as nanosecond and picosecond (150 ps and 35 ps) lasers, were employed to measure mercury and iodine using the LIBS and LB-TOFMS systems. Figure 12 shows LB-TOFMS and LIBS results of mercury measurement employing different pulse width lasers at different pressure. In the LIBS measurement of mercury in air, the value of I_Hg_/I_NO_ measured using 150 ps laser irradiation at 2000 Pa was set as 1 to evaluate the pulse width influence at different pressure. The ratios of I_Hg+_/I_N+_ and I_Hg_/I_NO_ can be improved using short pulse width lasers due to the larger ionization and excitation of Hg signal in multi-photon ionization process. Figure 13 shows LIBS measurement results of iodine under various conditions including pressure, laser power, wavelength, and pulse width. In order to evaluate the results of iodine measurement using the similar way, the value of I_I-183nm_/I_N_ measured using 150 ps laser irradiation at 2600 Pa was set as 1. The measurement results of short pulse width laser performed the improved intensity ratio of I_I-183nm_/I_N_. Taking all the conditions into consideration, the reasons for improved detection ability were the control of laser-induced plasma process, especially the electron impact ionization process, and the larger ionization and excitation of iodine.

The ratio of I_Hg+_/I_N+_ increased when increasing the pressure using ns and 35 ps lasers in LB-TOFMS. When the pressure increased in LIBS, the intensity ratios of I_Hg_/I_NO_ and I_I-183nm_/I_N_ decreased. These results lead to the conclusion that the detection ability of LB-TOFMS can be enhanced using short pulse width lasers at high pressure. Simultaneously the low pressure and short pulse LIBS offers remarkable detection features. Mercury and iodine were largely and efficiently ionized by short pulse width laser through the multi-photon ionization process, leading to the improved intensity of the mercury and iodine signals. After multi-photon ionization, the electron impact ionization process in LIBS can be controlled by short pulse laser irradiation, which reduced the interference of the coexisting molecular and atomic emissions at low pressure, such as NO emissions in mercury measurement and N emissions in iodine measurement when employing different buffer gases. In LB-TOFMS, the influence of electron impact ionization was not taken into account for the plasma generation of ion signals due to its low pressure conditions (less than 1 Pa).

### 4.6. Detection Limit

**Figure 14:**
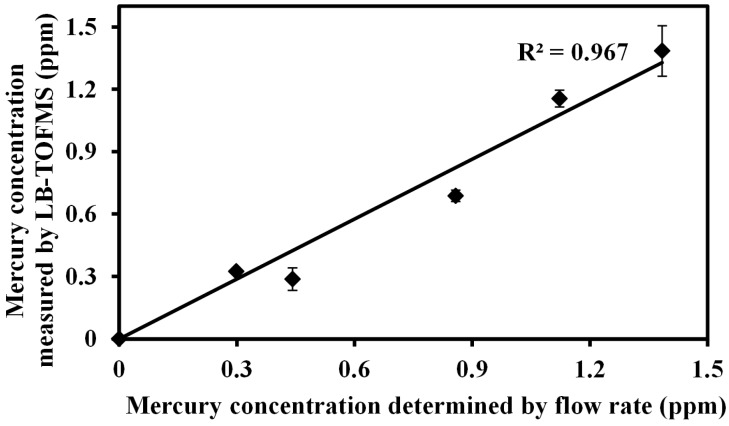
LB-TOFMS measurement of concentration dependence of Hg^+^ signal intensity using ns 532 nm laser irradiation. Conditions: buffer gas N_2_, power 110 mJ/p, pressure 0.056 Pa, lens angle α 10°.

Trace mercury and iodine species were measured using the LIBS and LB-TOFMS systems in this study. The concentration dependences in LIBS and LB-TOFMS display linear growth [59,67,71]. Figure 14 shows the concentration dependence of Hg^+^ signal intensity using nanosecond laser operated at 532 nm in LB-TOFMS. The detection limit of trace species using TOFMS is often ppb or less, which is much more sensitive than that of LIBS.

The detection limits of mercury and iodine measured by LIBS and LB-TOFMS are summarized in Table 4. In LIBS measurement of Hg in air, mercury detection limit of 600 shots (1 min) was estimated by subtracting the interference signal, which was mainly the NO(0,3) emission, from the measured LIBS spectra and it was calculated by evaluating the ratio of the slope of mercury calibration curve (ɛ) to background noise (standard deviation: σ) around 253.7 nm after subtraction of the interference signal. The detection limits of nanosecond and 35 ps laser irradiation were 450 ppb (3σ/ɛ) and 30 ppb (3σ/ɛ) at 700 Pa. According to the comparison of Figure 5b and Figure 6a, these results suggest that the signal to background ratio became worse in buffer gas of air due to the quenching of mercury signal in O_2_ and the NO emission effect on the background signal. Hence, the detection limit of mercury in N_2_ was enhanced to 3.5 ppb (3σ/ɛ) using nanosecond laser irradiation at 6600 Pa. In buffer gas of air, NO emissions can be reduced using short pulse width lasers to enhance the detection limit. The detection limit of mercury in N_2_ was not enhanced using 35 ps laser irradiation due to less effect of coexisting molecular and atomic emissions. The effective way to enhance the detection limit was to control the electron impact ionization process by means of reducing the pressure and to increase the mercury signal intensity by adopting the nanosecond laser irradiation when employing N_2_ as the buffer gas.

**Table 4:** Detection limits of Hg and iodine under different conditions.

	Species	LIBS (ppb)				LB-TOFMS (ppb)			
		Mercury		Iodine		Mercury		Iodine	
	**Conditions**	ns	35 ps	ns	35 ps	ns	35 ps	ns	35 ps
**Buffer Gas**		1064 nm	1064 nm	1064 nm	1064 nm	532 nm	532 nm	532 nm	532 nm
Air		450	30	2660	3440	---	---	---	---
N_2_		3.5	---	60	210	2.2	1.2	9.5	9.0

As for iodine measurement in the LIBS system at low pressure, the iodine detection limit of 600 shots (1 min) was estimated by evaluating the ratio of the slope of iodine calibration curve (ɛ) to background noise (standard deviation: σ) around 183 nm. The detection limit of iodine in N_2_ was 60 ppb (3σ/ɛ) using nanosecond laser irradiation at 700 Pa. The iodine detection limit was 210 ppb (3σ/ɛ) using 35 ps laser irradiation at 2000 Pa. In the measurement of iodine in N_2_, N emission at 174.3 nm was not an interference for the I(I) line at 183 nm. The signal intensity of iodine became important to enhance the detection limit. Therefore nanosecond laser irradiation provided satisfactory results for iodine measurement. Iodine was also measured in air to evaluate the buffer gas effect on the detection ability. The detection limit of iodine in air was high due to the reduction of the iodine signal by the high quenching rate of excited iodine in air. The detection limits of iodine were 2660 ppb (3σ/ɛ) at 700 Pa and 3440 ppb (3σ/ɛ) at 900 Pa using ns and 35 ps laser irradiation. Generally, when the coexisting molecular and atomic emissions become an interference with the signals, such as the NO(0,3) emission on the mercury signal, the method employing the short pulse width laser becomes useful to enhance the detection limit. However, the signal intensity was important for the enhanced detection limit in the case of iodine measurement in air. The detection limit of iodine in air can’t be enhanced using 35 ps laser irradiation.

In mercury and iodine measurements using the LB-TOFMS system, the detection limit of 600 shots (1 min) was estimated by evaluating the ratio of the slope of mercury and iodine calibration curve (ɛ) to background noise (standard deviation: σ) around *m/z* 200 and *m/z* 127. The detection limits of mercury and iodine using nanosecond laser irradiation were about 2.2 ppb (3σ/ɛ) and 9.5 ppb (3σ/ɛ) respectively, which can be enhanced to 1.2 ppb (3σ/ɛ) and 9.0 ppb (3σ/ɛ) using 35 ps laser irradiation due to the larger ionization and excitation of mercury and iodine signals in multi-photon ionization process. Mercury and iodine detection limits in air were consistent with those in N_2_. The detection limits were not affected by the buffer gases because of the non-collisional feature in LB-TOFMS, which is different in that aspect from LIBS. Comparing the detection ability of the LIBS and LB-TOFMS systems, the detection limits in air can be enhanced 25 times for mercury measurement and 295.6 times for iodine measurement using LB-TOFMS compared with that using LIBS. In N_2_ buffer gas, the detection limits can be enhanced 2.9 times for mercury measurement and 6.7 times for iodine measurement.

## 5. Conclusions

In this study laser-induced breakdown spectroscopy (LIBS) and laser breakdown time-of-flight mass spectrometry (LB-TOFMS) were employed to measure trace species containing the elements mercury, iodine, strontium, cesium and arsenic for industrial needs. The elements mercury and iodine were mainly studied to compare the detection features of the LIBS and LB-TOFMS systems. A laser beam was introduced to break and ionize the molecules and atoms in samples. After the production and growth of plasma, the emission and ion signals were detected using the LIBS and TOFMS systems respectively. To summarize, the following conclusions can be made:

(1) Clear emission signals of mercury, iodine, strontium and cesium with decreased interference of the continuum emission from the plasma itself were measured using LIBS at low pressure. As for LB-TOFMS, the measurement results of 1064 nm and 532 nm laser irradiation show clear ion signals of arsenic, mercury and iodine without any interference of the partial fragmentation in the *m/z* 30–300 mass region.
(2) When the pressure decreased in low pressure LIBS, the ratios of I_Hg_/I_NO_ and I_I-183nm_/I_N_ increased due to the control of the electron impact ionization process. In TOFMS the intensity of Hg^+^ and I^+^ signals increased when the pressure increased due to the increased ion number density. In both detection systems, the measurements employing 532 nm laser irradiation provided improved results relating to the high photon energy concerning the multi-photon ionization process.
(3) The laser power dependence of the Hg^+^ signal shows an increase first and then a decrease because of the recombination of Hg^+^ ion with electrons. The intensity of the Hg emission signal increased when the laser power increased. In LIBS the measured result of signal to background ratio at 400 mJ/p was much better than that at 1000 mJ/p due to the decline of the electron impact ionization effect. An identical tendency was observed in iodine measurement.
(4) The delay time is an important parameter in the LIBS process concerning the plasma temperature. Under low pressure conditions, the delay time was not a determining factor for mercury measurements in air. In the measurement of iodine using 532 nm laser irradiation, I_I-183nm_/I_N_ increased when the delay time increased. This can be explained by the different upper level energy.
(5) The laser-induced plasma process can be also controlled by a short pulse width laser, especially the electron impact ionization process, as well as the larger ionization and excitation of mercury and iodine signals during the multi-photon ionization process. The detection ability of trace species can be enhanced using short pulse width laser in LIBS and LB-TOFMS.
(6) The detection ability was improved dramatically using the LB-TOFMS system. The detection limits of mercury and iodine in N_2_ were 3.5 ppb (3σ/ɛ) and 60 ppb (3σ/ɛ) using low pressure LIBS. Mercury and iodine detection limits using of LB-TOFMS were 1.2 ppb (3σ/ɛ) and 9.0 ppb (3σ/ɛ), which were enhanced 2.9 times for mercury measurement and 6.7 times for iodine measurement, respectively, compared with that using LIBS because of the different detection features.

The detection features of trace species using laser-induced breakdown spectroscopy (LIBS) and laser breakdown time-of-flight mass spectrometry (LB-TOFMS) were exhaustively compared by trace species measurements of mercury and iodine. These detection systems with different features can be employed for various applications according to the conditions of each application.
